# Sulfiredoxin as a Potential Therapeutic Target for Advanced and Metastatic Prostate Cancer

**DOI:** 10.1155/2020/2148562

**Published:** 2020-01-20

**Authors:** Caroline N. Barquilha, Nilton J. Santos, Caio C. D. Monção, Isabela C. Barbosa, Flávio O. Lima, Luis A. Justulin, Nelma Pértega-Gomes, Sérgio L. Felisbino

**Affiliations:** ^1^Department of Morphology, Institute of Biosciences, São Paulo State University, Botucatu, 18618689 SP, Brazil; ^2^Institute of Biology, State University of Campinas, Campinas, 13083862 SP, Brazil; ^3^Department of Pathology, Botucatu Medical School, São Paulo State University, Botucatu, 18618687 SP, Brazil; ^4^Department of Medical Oncology, Dana-Farber Cancer Institute, Harvard Medical School, Boston, 02215 MA, USA

## Abstract

The incidence of prostate cancer (PCa) is increasing, and it is currently the second most frequent cause of death by cancer in men. Despite advancements in cancer therapies, new therapeutic approaches are still needed for treatment-refractory advanced metastatic PCa. Cross-species analysis presents a robust strategy for the discovery of new potential therapeutic targets. This strategy involves the integration of genomic data from genetically engineered mouse models (GEMMs) and human PCa datasets. Considering the role of antioxidant pathways in tumor initiation and progression, we searched oxidative stress-related genes for a potential therapeutic target for PCa. First, we analyzed RNA-sequencing data from *Pb-Cre4; Pten^f/f^* mice and discovered an increase in sulfiredoxin (*Srxn1*) mRNA expression in high-grade prostatic intraepithelial neoplasia (PIN), well-differentiated adenocarcinoma (medium-stage tumors), and poor-differentiated adenocarcinoma (advanced-stage prostate tumors). The increase of SRXN1 protein expression was confirmed by immunohistochemistry in mouse prostate tumor paraffin samples. Analyses of human databases and prostate tissue microarrays demonstrated that SRXN1 is overexpressed in a subset of high-grade prostate tumors and correlates with aggressive PCa with worse prognosis and decreased survival. Analyses in vitro showed that *SRXN1* expression is also higher in most PCa cell lines compared to normal cell lines. Furthermore, siRNA-mediated downregulation of SRXN1 led to decreased viability of PCa cells LNCaP. In conclusion, we identified the antioxidant enzyme SRXN1 as a potential therapeutic target for PCa. Our results suggest that the use of specific SRXN1 inhibitors may be an effective strategy for the adjuvant treatment of castration-resistant PCa with SRXN1 overexpression.

## 1. Introduction

The incidence of prostate cancer (PCa) has progressively increased in the western world, representing the second most prevalent cancer with the second highest mortality rate in men [1–3]. Androgen receptor (AR) and circulating androgen are essential for normal prostate development [4], and AR is the main oncogenic driver of PCa initiation and progression. Therefore, therapeutic strategies against this type of tumor are usually aimed at inhibiting AR activity [5, 6]. If detected early, the chances of curing PCa are high, but more advanced PCa develops resistance to androgen deprivation therapies [7, 8]. These tumors are referred to as “castration-resistant PCa,” are highly heterogeneous in their molecular alterations [9–11], and are resistant to available therapies [12–14]. It is therefore crucial to identify new therapeutic targets and additional approaches to cure or at least increase the survival of patients with advanced PCa [15, 16].

Emerging technologies have allowed a deeper understanding of the cancer genome and the differential expression of genes involved in tumor development [17–19]. One recent example of the success of modern precision medicine for the treatment of cancer is the development of PARP1 inhibitors (PARPi). This specific adjuvant treatment benefits patients with defective DNA-damage repair such as the *BRCA1* and *BRCA2* mutations that frequently occur in breast and ovarian cancers [20, 21]. Studies involving PARPi for the treatment of PCa are already underway [22, 23]. In this context, one strategy for the discovery of new cancer biomarkers and/or potential therapeutic targets is to integrate genomic data from genetically engineered mouse models (GEMMs) and human cancer patients [24, 25]. Data generated by gene expression analysis of GEMM tumors help to search through genome-wide expression datasets generated from human prostate tumors. GEMMs are also indispensable in preclinical studies to test new drugs in immunocompetent animals [26]. Thus, cross-species analyses provide a powerful tool to pinpoint genes conserved across both species that are master regulators of tumor development [27–29].

Cancer is a complex disease involving many molecular variables. For PCa, oxidative stress is one of the main age-associated factors that influences the risk of developing this tumor [30, 31], such as alterations in *GSTP1* expression by hypermethylation [32]. Several studies suggest that the prostate is exceptionally vulnerable to elevated reactive oxygen species (ROS) and oxidative stress [33, 34]. In normal cells, elevated ROS causes cumulative damage in lipids, proteins, and DNA, which may result in mutations and cancer initiation [35–37]. However, oxidative stress is also directly involved in cancer progression and metastasis [38–40]. Antioxidant pathways play an important cytoprotective role in tumors by preventing treatment-induced apoptosis and conferring chemoresistance [41–45]. Since these tumor cells are highly dependent on antioxidant mechanisms [38, 40], we aimed to identify genes involved in oxidative stress homeostasis that can be therapeutically targeted for the treatment of PCa. Using cross-species analyses of GEMMs and human data, we have investigated antioxidant genes with altered expression in different stages of PCa progression. In this study, we identified the antioxidant enzyme sulfiredoxin (SRXN1) as a potential target, and we validated its relevance in advanced PCa.

## 2. Materials and Methods

### 2.1. Genetically Engineered Mouse Model (GEMM)

We used RNAseq data and prostate samples from GEMM *Pten^f/f^*, wild type (WT), and from the *Pb-Cre4; Pten^f/f^*, which contains a deletion in both alleles of *Pten*, a tumor suppressor gene, exclusively in the prostate epithelium (conditional knockout). This model shows stages of tumor progression similar to human PCa, such as prostatic intraepithelial neoplasia (PIN), microinvasive and invasive well-differentiated adenocarcinoma (medium-stage tumors, MT), and fully invasive poorly differentiated adenocarcinoma (advanced-stage tumors, AT), with slow progression pace. Moreover, PTEN loss has been consistently associated with more aggressive disease features and worse prognosis, since PTEN loss range from less than 20% in clinically localized prostate tumors to more than 40% in metastatic castration-resistant PCa [46]. Although there are other interesting GEMMs for PCa, few studies have combined the stages of tumor progression with all prostatic lobes (anterior, AP; ventral, VP; lateral, LP; and dorsal prostate, DP) in a deep RNA-sequencing experiment. Additional details about this conditional knockout mice, histopathological analysis, and transcriptome data have been previously described [29].

RNA-sequencing data from samples of all prostatic lobes were accessed through the *NCBI Gene Expression Omnibus* (GEO, https://www.ncbi.nlm.nih.gov/geo/) platform, reference number GSE94574. Briefly, 72 samples were submitted to RNAseq analysis, including 20 WT prostatic lobes, 16 PIN, 20 MT, and 16 AT. At least four samples for each prostatic lobe and pathological condition were submitted to RNAseq analysis. First, we looked for the differentially expressed genes in each lobe and stages of tumor progression with adjusted *p* value < 0.05. We filtered all the deregulated genes using a known list of 84 genes involved in oxidative stress response [47, 48] and selected those which were altered in at least three prostatic lobes in PIN, MT, and/or AT. To select a potential therapeutic target, the consensus list of the upregulated genes involved in oxidative stress pathways was evaluated for clinical relevance in PCa using the *cBioPortal for Cancer Genomics* [49, 50], *Cambridge Carcinoma of the Prostate App* (CamcAPP) [51], and *SurvExpress* [52] databases.

The animal experiments used in this study were approved by the CRUK Institute Ethics Committee of the Cambridge University, UK, under design license 80/2435 and by the Ethics Committee on Animal Experimentation from the Institute of Biosciences of Botucatu, UNESP, Brazil, under protocol CEEA 613/2014.

### 2.2. Immunohistochemistry (IHC)

Paraffin blocks of all prostatic lobes containing WT and tumor samples (PIN, MT, and AT) from GEMMs were obtained by donation from David Neal's Uro-Oncology Group at the CRUK Cambridge Institute (University of Cambridge, UK). Histological sections (5 *μ*m) of WT and tumor-bearing prostate lobes from the GEMMs (*n* = 5 per group) were deparaffinized, rehydrated, boiled for 30 min in 10 mM sodium citrate solution (pH 6.0) for antigen retrieval, and quenched in 3% H_2_O_2_ methanol solution. Prostate sections were blocked in 5% nonfat milk in phosphate-buffered saline (PBS) and incubated overnight at 4°C with a specific primary antibody against SRXN1 (Abcam, ab92298, 1 : 100), our chosen gene. Next, sections were incubated with a secondary peroxidase-conjugated antibody (Santa Cruz Biotechnology, 1 : 200), which was developed using diaminobenzidine (Sigma-Aldrich) as the chromogen. Slides were counterstained with Harris's hematoxylin. The negative control was obtained by excluding the primary antibody incubation step. The sections were visualized using a Leica DMLB 80 microscope.

### 2.3. Human Database Analyses

The antioxidant enzyme SRXN1 was chosen as a potential target by RNA-sequencing and IHC analysis of the prostate tumors from GEMMs. To validate the SRXN1 expression pattern in human PCa, we searched available databases from published studies. We used *GEO* (https://www.ncbi.nlm.nih.gov/geoprofiles/) to profile *SRXN1* gene expression from prostate tumors of different grades, and from different normal and epithelial prostate tumor cell lines. We also investigated the *SRXN1* gene expression pattern using the *cBioPortal for Cancer Genomics* database (http://www.cbioportal.org/), using studies from *The Cancer Genome Atlas*, TCGA (https://cancergenome.nih.gov/), the *CamcAPP* dataset (https://bioinformatics.cruk.cam.ac.uk/apps/camcAPP/), and the *SurvExpress* database (http://bioinformatica.mty.itesm.mx:8080/Biomatec/SurvivaX.jsp) to determine the association of *SRXN1* gene alterations with patient clinical data, such as risk/prognosis and survival rates.

### 2.4. Human Prostate Tumor Tissue Microarrays (TMAs)

After validating the overexpression of SRXN1 in GEMM samples and human database analyses, we investigated the SRXN1 protein expression in human prostate samples. Human TMAs were constructed using the prostates of patients who underwent radical prostatectomy between 1980 and 2000. Of the patients, 104 samples originated from organ-confined tumors, and 16 samples originated from adjacent nonneoplastic tissue. One-tissue cores of 1 mm diameter were used for each sample. The TMA was donated to and analyzed by the consultant pathologist Flávio de Oliveira Lima at the Botucatu Medical School, UNESP, Brazil. This study was approved by the Medical Ethics Committee of the Botucatu Medical School, UNESP, Brazil (protocol number 3888/2011).

According to glandular histoarchitecture and histopathological staging, tumor samples from human TMAs were classified into five prognostic categories (1-5, from more differentiated to less differentiated), according to the Gleason score [53, 54] and the International Society of Urological Pathology (ISUP) grade [55, 56]. The prostate tumor classifications from TMAs are presented in Table 1.

SRXN1 protein expression in human TMAs was detected by immunohistochemistry following the previously described protocol. The results were quantified and evaluated as negative (no staining, score 0) or positive (staining present, score 1), independent of the staining intensities. This analysis was performed by two independent observers without access to clinical data. SRXN1 staining scores were associated with the clinical and pathological characteristics (Gleason score, prognosis category, and survival time). Details regarding the clinical data and the SRXN1 IHC score are available in Supplementary Table S1, and the association between Gleason score/prognosis and survival rates in Supplementary Figure S1.

### 2.5. Cell Lines and Culture Conditions

The following three prostate cell lines were used for the analyses: RWPE-1 (normal), LNCaP (tumor, androgen sensitive), and PC-3 (tumor, castration-resistant). The cells were obtained from the American Type Cell Culture (Manassas, Virginia, USA). LNCaP and PC-3 cell lines were cultured using RPMI 1640 medium supplemented with 2 mM L-glutamine, 10% fetal bovine serum, 50 *μ*g/mL penicillin, 50 *μ*g/mL streptomycin, and 0.5 *μ*g/mL amphotericin B (GIBCO/Invitrogen). RWPE-1 cells were cultured with a Keratinocyte Serum Free Medium Kit supplemented with bovine pituitary extract and recombinant human epidermal growth factor (GIBCO/Invitrogen). The medium was changed twice per week, and the cells were monitored daily under an inverted microscope (Zeiss Axiovert). Cells were grown at 37°C and 5% CO_2_. For passaging, cells were detached with 0.25% trypsin (GIBCO/Invitrogen) for 5 min at 37°C, resuspended in a growth medium, and reseeded. Cell lines were authenticated by Short Tandem Repeat (STR) DNA profiling by the Biorepository Facility of the Institute of Biosciences, UNESP, Brazil. Mycoplasma testing was carried out at regular intervals throughout the experiments, and the results were negative.

### 2.6. RNA Extraction and RT-qPCR

Total RNA from prostate cell lines RWPE-1, LNCaP, and PC-3 was extracted using the RNeasy mini kit (QIAGEN) according to the manufacturer's instructions. RNA quantification was determined by a Nanovue Spectrophotometer (GE Healthcare). cDNA was synthesized using the High-Capacity cDNA Reverse Transcription Kit (Applied Biosystems). qRT-PCR reactions were performed using the QuantStudio 12K Flex Real-Time PCR system (Applied Biosystems). Relative gene expression was calculated using the 2^-*ΔΔ*CT^ method [57]. The gene target detected was *SRXN1*, and *β*-actin (*ACTB*) was used as a housekeeping gene. Details of primers used are given in Table 2.

### 2.7. Sulfiredoxin Knockdown *In Vitro*

To analyze the effects of SRNX1 suppression *in vitro*, we chose PCa cell line LNCaP as our model due to its elevated SRXN1 mRNA expression. LNCaP cells (1 × 10^5^) were seeded in 6-well plates using a complete RPMI medium. The transfection was performed using the Lipofectamine RNAi MAX Transfection Reagent (Invitrogen) according to the manufacturer's instructions. Lipofectamine and siRNA targeting the mRNA of *SRXN1* (MISSION esiRNA, Sigma-Aldrich) were individually diluted in an Opti-MEM medium (GIBCO/Invitrogen), mixed, and incubated for 5 min. Next, siRNA-lipid complex was added to cells and incubated for 48 h. The final siRNA concentration was 25 and 50 nM. In addition, siRNA targeting eGFP (MISSION esiRNA, Sigma-Aldrich) was used as negative control.

### 2.8. Cell Viability Assays

LNCaP cells (6 × 10^4^) were seeded in 24-well plates. Once cells became 60% confluent, siRNA against the mRNA of *SRXN1* was added as previously described. After 48, 72, and 96 h of transfection, cell viability was determined by the MTT (Thiazolyl Blue Tetrazolium Bromide, Sigma-Aldrich) reduction method according to the manufacturer's instructions [58, 59]. The reaction was transferred to a 96-well plate, and the absorbance (550 nm) was read by a spectrophotometer (ASYS HITECH GmbH, Eugendorf) to determine the percentage of cell viability relative to control cells.

### 2.9. Statistical Analysis

For parametric data, Student's *t*-test with Welch's Correction Factor or Factorial ANOVA was used. For nonparametric data, the Mann-Whitney or Kruskal-Wallis test was used. For association analyses, the Chi Square Contingency test and Kaplan-Meier/Log-Rank test were used. Differences were considered statistically significant when *p* < 0.05. Statistical analyses were performed using the GraphPad Prism program (version 5.0).

## 3. Results

### 3.1. Oxidative Stress Response Genes Are Deregulated during Prostate Tumor Progression in *Pb-Cre4; Pten^f/f^* Mice

**Figure 1:**
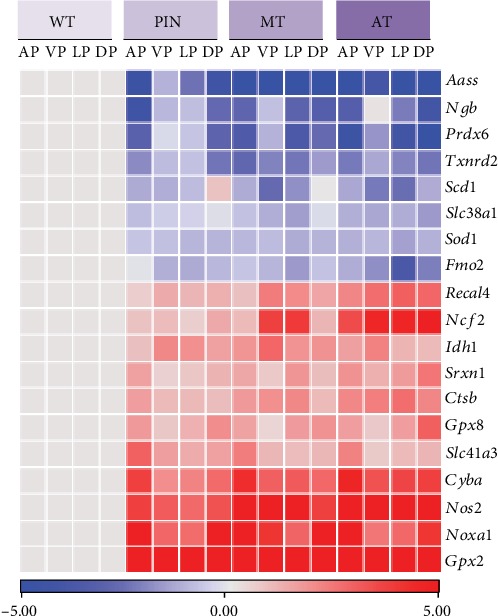
Consensus deregulated oxidative stress genes in prostate tumors from *Pb-Cre4; Pten^f/f^* mice. Heat map of oxidative stress response genes in the four lobes (anterior, AP; ventral, VP; lateral, LP; and dorsal prostate, DP) of wild-type (WT) prostate and tumor samples (prostatic intraepithelial neoplasia, PIN; medium-stage tumors, MT; and advanced-stage tumors, AT) from GEMM *Pb-Cre4; Pten^f/f^*. Relative gene expression level (median) is showed as log_2_ fold change related to WT. Values between -5 and 0 represent downregulated genes (blue gradient) and between 0 and 5 upregulated genes (red gradient).

RNAseq data from GEMMs showed on average 2,000 genes differently expressed in each prostatic lobe (AP, VP, LP, and DP) at the tumor stages of PIN, MT, and AT. We filtered all these deregulated genes using a known list of 84 genes involved in oxidative stress response [47, 48]. We selected those genes which were changed in at least three prostatic lobes in PIN, MT, and/or AT, resulting in a final list with the top 19 deregulated oxidative stress-related genes, out of which 11 upregulated and 8 downregulated (Figure 1). We investigated the prognosis significance of the upregulated and druggable genes. Excluding those genes with no clinical relevance, and those which have already been associated with PCa, such as *Ctsb*, *Gpx2*, *Idh1*, and *Nos2* [60–66], we selected the antioxidant enzyme *Srxn1* that had a strong correlation with patient outcome and has no previous related functional studies in PCa.

### 3.2. *SRXN1* Expression Is Increased in Prostate Tumors from *Pb-Cre4; Pten^f/f^* Mice

**Figure 2:**
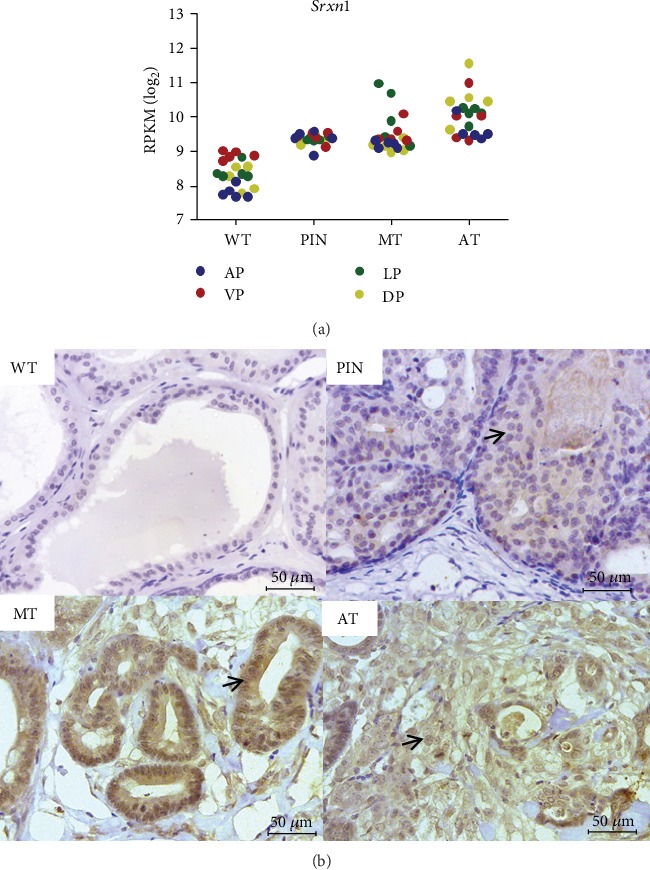
SRXN1 gene and protein expression is higher in prostate tumor tissue from *Pb-Cre4; Pten^f/f^* mice compared to normal tissue. (a) *Srxn1* expression levels in the four lobes (anterior, AP; ventral, VP; lateral, LP; and dorsal prostate, DP) of wild-type (WT) prostate and tumor samples (prostatic intraepithelial neoplasia, PIN; medium-stage tumors, MT; and advanced-stage prostate tumors, AT) from *Pb-Cre4; Pten^f/f^* mice. Data are expressed as log_2_ of reads per kilobase per million (RPKM). The relative curve of *Srxn1* mRNA expression increases significantly with *p* < 0.001. (b) Representative images of immunohistochemistry detecting SRXN1 protein in WT and prostate tumors in different stages of progression: PIN, MT, and AT from *Pb-Cre4; Pten^f/f^* mice. Scale bars = 50 *μ*M. Arrows indicate positively stained cells.

Prostate transcriptome data from *Pb-Cre4; Pten^f/f^* mice comparing WT to tumor samples (PIN, MT, and AT) showed that the relative mRNA expression of *Srxn1* increases progressively and significantly during tumor progression in all prostatic lobes, with *p* < 0.001 (Figure 2(a)). To evaluate the pattern of SRXN1 protein expression in PCa, IHC analysis was performed, and we observed increased SRXN1 protein expression (higher intensity immunostaining) in prostate tumors compared to WT tissue (Figure 2(b)).

### 3.3. *SRXN1* Is Overexpressed in High-Grade Human Prostate Tumors and Is Associated with Cancer Aggressiveness

**Figure 3:**
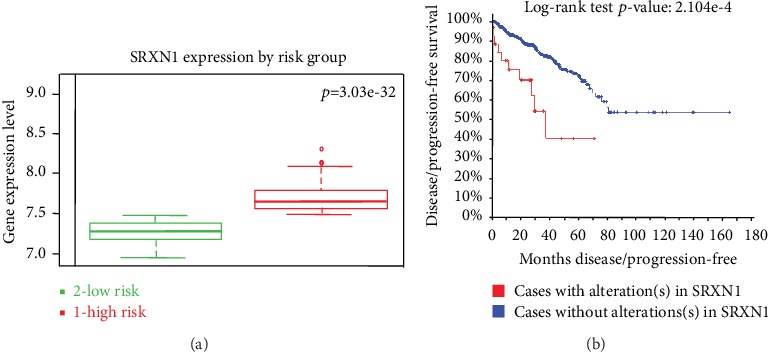
The increased expression of *SRXN1* in patients with prostate cancer (PCa) is associated with high-risk/worse prognosis and lower disease-/progression-free survival. (a) The level of *SRXN1* gene expression (median) in PCa patients with low-risk/better prognosis (green) and patients with high-risk/worse prognosis (red). Data and analyses were cataloged using the SurvExpress database [52] from a MSKCC study [18]. The difference between boxplots is statistically significant with *p* = 3.03^−32^. (b) Kaplan-Meier curve displaying disease-/progression-free survival of PCa patients with (red) or without (blue) *SRXN1* alterations, cataloged using the cBioPortal human database [49, 50] from a provisional study of TCGA (https://cancergenome.nih.gov/). Curves are significantly different with *p* = 2.104^−4^.

Published and available data (database GEO profiles) on global gene expression in human PCa demonstrated increased expression of *SRNX1* in patients with advanced tumors (Gleason scores 8 and 9) relative to control (Supplementary Figure S2a). Similarly, studies available in the SurvExpress database revealed that increased expression of *SRXN1* correlates with a high-risk/worse prognosis of patients with PCa (Figure 3(a)). The same association was observed using several studies available in the CamcAPP dataset. This resource demonstrated an increase in *SRXN1* expression in clusters of patients with poor prognosis (Supplementary Figure S2c, S2d, and S2e). Data from TCGA studies obtained from cBioPortal demonstrated that patients with alterations in *SRXN1* (especially overexpression) have a lower disease-free/progression-free survival (Figure 3(b)). The same effect was observed using the CamcAPP dataset (Supplementary Figure S2f).

### 3.4. SRXN1 Protein Expression Is Associated with Poor Prognosis and Lower Survival of PCa Patients

**Figure 4:**
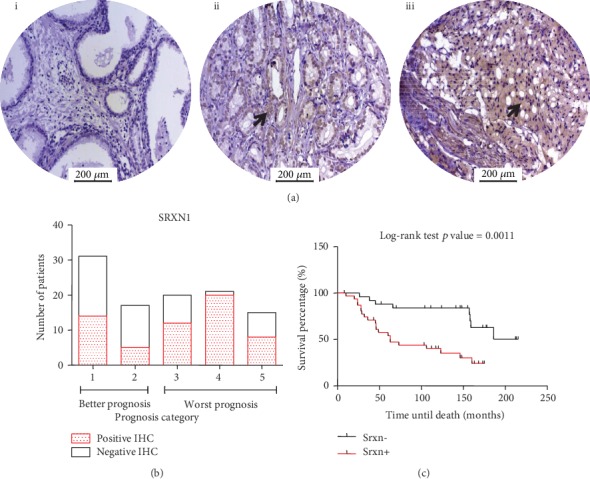
SRNX1 protein expression is increased in human advanced prostate cancer (PCa) and is associated with worse prognosis and decreased survival. (a) Representative image of SRXN1 immunohistochemistry (IHC) in (i) adjacent nonneoplastic tissue, (ii) medium-stage/low-grade tumor, and (iii) advanced-stage/high-grade tumor from tissue microarrays (TMAs) of human prostate samples. Arrows indicate positively stained cells. Scale bars = 200 *μ*M. (b) Representative graph showing the association between SRXN1 protein expression (by IHC analysis in human prostate TMAs) and prognosis, which were divided into groups with good prognosis (categories 1 and 2) and worse prognosis (categories 3, 4, and 5), according to the International Society of Urological Pathology (ISUP) grade. White bars represent patients with negative SRXN1 immunostaining, and red bars represent patients with positive immunostaining. (c) Global survival curve of patients with PCa obtained from SRXN1 IHC analyses in human prostate samples (TMAs) associated with patient survival data. The Kaplan-Meier curve from patients with negative SRXN1 immunostaining is represented by black (Srxn-) and positive immunostaining by red (Srxn+). Curves are significantly different with *p* = 0.0011.

To further validate the association of SRXN1 with PCa aggressiveness, we performed IHC analyses on SRXN1 in TMAs of human prostate samples (benign and cancerous tissue). Protein expression was increased (positive immunostaining) in more than 70% of high-grade tumors (Figure 4(a)), representing patients with high Gleason score and worse prognosis.

Additionally, the negative and positive immunostaining of SRNX1 in TMAs was associated with clinical data. It was observed that among the 48 patients with better outcome (prognosis categories 1 and 2), 39.5% expressed SRXN1. In comparison, among the 56 patients with worse prognosis (categories 3, 4, and 5), 71.4% expressed SRXN1, which differed significantly (*p* = 0.0015) (Figure 4(b)). Analysis of survival time (*n* = 69) revealed an association between SRXN1 protein expression and decreased survival (Figure 4(c)). Interestingly, the expression pattern of SRXN1 stratified patients as well as the prognosis score according to the ISUP classification, since patients with expression of SRXN1 had decreased survival similarly to patients with worse prognosis (Supplementary Figure S1).

### 3.5. PCa Cell Lines Express Higher Levels of *SRXN1* Compared to Normal Cell Lines, and Inhibition of SRXN1 mRNA in the PCa Cell Line LNCaP Decreases Viability

**Figure 5:**
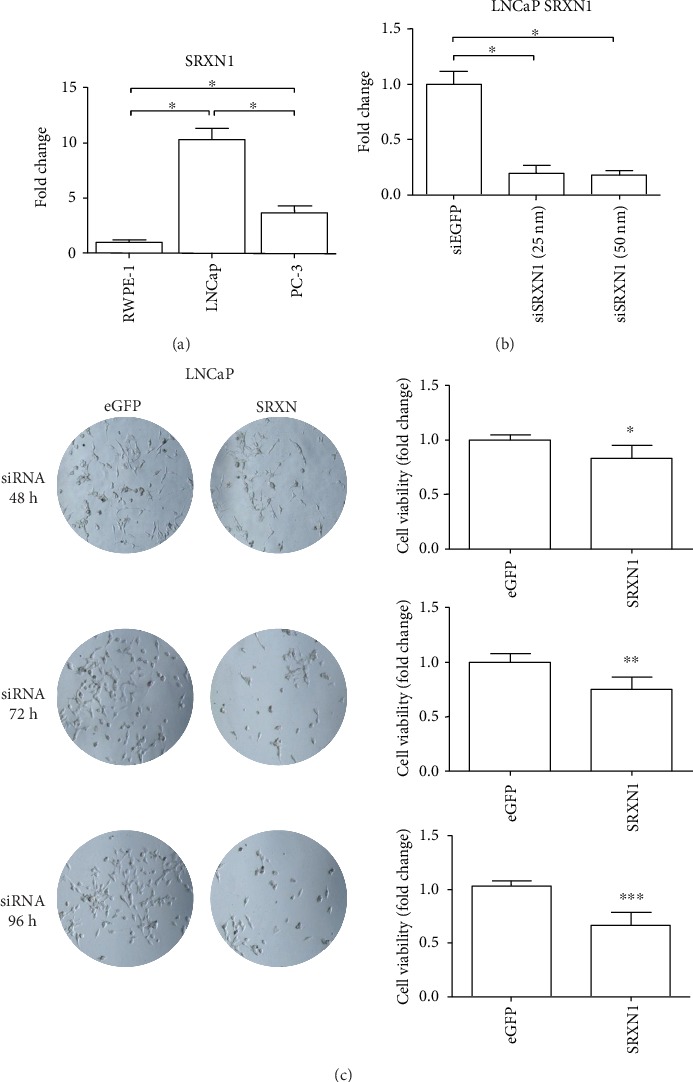
Prostate tumor cell lines overexpress *SRXN1*, and attenuation of *SRXN1* mRNA levels in prostate cancer (PCa) cell line LNCaP decreases tumor cell viability. (a) *SRXN1* gene expression level in prostate cell line RWPE-1 (normal), LNCaP (PCa, androgen sensitive), and PC-3 (PCa, castration-resistant). ^∗^Denotes statistical significance with *p* < 0.0001. Data are expressed as fold change normalized to *ACTB* expression. (b) qRT-PCR graph of *SRXN1* gene expression in LNCaP cells after transfection (48 h) with siRNA targeting eGFP (control) or *SRXN1* mRNA (25 and 50 nM). ^∗^Denotes statistical significance with *p* < 0.0001. (c) Cell viability of PCa cell line LNCaP without (control, eGFP) and with inhibition of *SRXN1* mRNA by siRNA-mediated silencing (25 nM) after 48, 72, and 96 h. ^∗^Denotes statistical significance with *p* = 0.0043; ^∗∗^*p* = 0.0002; and ^∗∗∗^*p* < 0.0001.

Quantitative PCR showed that prostate tumor cell lines LNCaP and PC-3 express higher levels of *SRXN1* than the normal prostate cell line RWPE-1 (Figure 5(a)). Among the prostate tumor cell lines, LNCaP has increased *SRXN1* gene expression compared to PC-3 (Figure 5(a)). Database analysis of other studies from GEO profiles also showed increased expression of *SRXN1* in LNCaP and PC-3, and in most prostate tumor cells (Supplementary Figure S2b).

To analyze the effects of decreased SRXN1 expression *in vitro*, we performed siRNA-mediated knockdown of *SRXN1* mRNA followed by cell viability assays at three different time points after transfection (48, 72, and 96 h). PCa cell line LNCaP was chosen because of its high expression of *SRXN1*. After transfection, we observed a >80% reduction of the *SRXN1* mRNA (Figure 5(b)). The silencing analyses demonstrated that the viability of LNCaP cells with decreased *SRXN1* mRNA expression was lower compared to cells that expressed *SRXN1* (eGFP siRNA, negative control). The viability of PCa cells significantly decreased in all analyzed time points after *SRXN1* knockdown (Figure 5(c)) (*p* < 0.05 in all cases).

## 4. Discussion

Cross-species analyses from GEMMs and human data revealed that SRXN1 is overexpressed in PCa tissue and cell lines, with a remarked increase in advanced tumors. Cancer cells are more dependent on antioxidant mechanisms and more vulnerable to ROS-induced damage than normal cells [38, 40]. SRXN1 is an antioxidant enzyme induced by NRF2 and AP-1 and plays an important role in oxidative stress balance [67–69]. Specifically, SRXN1 acts in peroxiredoxin (I-IV) reactivation [70]. The peroxiredoxins are a group of peroxidase enzymes responsible for the reduction of ROS, such as hydrogen peroxide and organic peroxides [71], protecting the cell from high levels of ROS-induced oxidative stress. By reducing the hyperoxidized peroxiredoxins, SRXN1 protects them from degradation [72].

Several studies have demonstrated the protective role of SRXN1 against oxidative injury [73, 74]. The increased expression of SRXN1 has been observed especially in solid tumors, as we observed for PCa in this study. Wei *et al*. showed elevated SRXN1 protein expression in lung tumor samples by IHC analyses [75]. Another study from Wei *et al*. demonstrated high SRXN1 protein expression in human colon carcinoma and showed that *Srxn1* knockout animals were resistant to carcinogenic induction [76]. In addition, *SRXN1* expression is increased in different human skin malignancies [77, 78] and gastric cancer [79].

In addition to the overexpression of SRXN1 in PCa high-grade tumors, the current study also showed a close relationship between SRXN1 expression and both cancer aggressiveness and patient outcome. Researchers have already demonstrated that SRXN1 is essential for cancer cell proliferation [80], and its depletion decreases cell viability [74], suppresses cell migration, and inhibits tumor growth [75, 77], increasing the aggressiveness of cancers with *SRXN1* upregulation. Polymorphisms in the *SRXN1* gene have been shown to promote breast cancer development and influence patient survival [81]. *SRXN1* expression is also associated with poor survival of patients with pancreatic adenocarcinoma [82]. Raatikainen *et al*. observed an inverse correlation between *SRXN1* expression and worse PCa prognosis [83], in contrast to our results and the results of other studies.

Considering the pathogenic role of SRXN1 in human cancer, it has already been suggested as a new potential therapeutic target for different tumors, but not yet for PCa. In our study, we observed that decreasing *SRXN1* mRNA in PCa cell line LNCaP decreased cell viability, reinforcing the importance of this antioxidant enzyme in PCa cells and suggesting that SRXN1 activity supports tumor cell survival and growth. In malignant human skin tumors, Wei *et al*. showed that *SRXN1* inhibition (by the AP-1 pathway) may be a novel strategy for skin cancer prevention and treatment [77]. Kim *et al*. and Kim *et al*. demonstrated that *SRXN1* inhibition by synthetic inhibitors (J14 and K27) selectively promotes the death of A549 pulmonary tumor cells [84, 85]. All these studies suggest that when SRXN1 activity is inhibited, the high ROS levels exceed cell antioxidant capacity, resulting in accumulated damage that selectively leads to tumor cell death. Thus, our work proposes that SRXN1 can be an interesting therapeutic target for further preclinical *in vivo* tests using an immunocompetent and clinically relevant GEMM. Patients with advanced PCa presenting SRXN1 overexpression may benefit from SRXN1 inhibition therapy, providing cancer patients with more personalized treatment.

## 5. Conclusions

SRXN1 is increased in a subset of PCa patients with high-grade tumors (advanced stage) and correlates with poor prognosis and worse survival. Thus, our cross-species analyses pinpoint SRXN1 as a potential therapeutic target for PCa, which plays an important role in protection of prostate tumor cells against oxidative stress. We hypothesize that the use of selective SRXN1 inhibitors can be an effective adjuvant treatment strategy for metastatic PCa with SRXN1 overexpression.

## Figures and Tables

**Table 1:** Number of cases and tumor classification (Gleason score and prognosis category) from human prostate samples (tissue microarrays).

Number of cases	Gleason score	Prognosis category
16	Adjacent nonneoplastic tissue	—
31	6 (3 + 3)	1
17	7 (3 + 4)	2
20	7 (4 + 3)	3
1	8 (3 + 5)	4
19	8 (4 + 4)	
1	8 (5 + 3)	
6	9 (4 + 5)	5
5	9 (5 + 4)	
4	10 (5 + 5)	

**Table 2:** Primers used in the RT-qPCR reactions.

Genes	Primer forward	Primer reverse	Amplicon
*SRXN1*	CAAGGTGCAGAGCCTCGT	CAGCCCCCAAAGGAGTAGAA	105
*ACTB*	GATTCCTATGTGGGCGACGA	TGTAGAAGGTGTGGTGCCAG	124

## Data Availability

The RNAseq data from the GEMM *Pb-Cre4; Pten^f/f^* mouse used to support the findings of this study have been deposited in the NCBI Gene Expression Omnibus repository (https://www.ncbi.nlm.nih.gov/geo/), reference number GSE94574. Previously reported human databases were used to support this study and are available at the NCBI Gene Expression Omnibus (https://www.ncbi.nlm.nih.gov/geoprofiles/), the cBioPortal for Cancer Genomics (http://www.cbioportal.org/), The Cancer Genome Atlas (TCGA) (https://cancergenome.nih.gov/), the Cambridge Carcinoma of the Prostate App (camcAPP dataset) (https://bioinformatics.cruk.cam.ac.uk/apps/camcAPP/), and the SurvExpress database (http://bioinformatica.mty.itesm.mx:8080/Biomatec/SurvivaX.jsp). These prior studies (and datasets) are cited at relevant places within the text as references [18, 19, 49–52] and Satake *et al.* (supplementary material [1]) and Zhao *et al.* (supplementary material [2]). The clinical data of the PCa patients from TMA samples used to support the findings of this study are included within the supplementary information files (Table S1).

## Supplementary Materials

Supplementary Materials Table S1: description of the patient clinical data used in the preparation of the TMAs, such as Gleason score, prognostic category, survival time, and patient outcome. Figure S1: patients with PCa grouped by prognosis category (1-5) show expected survival curves. (A) Global survival curve of patients with PCa associated to prognosis category (1-5, from more differentiated to less differentiated), according to the ISUP grade. Clinical data are from those patients which prostate samples were used to construct TMAs. Kaplan-Meier curves are statistically different with *p* < 0.0001. (B) Additional analysis of the same patients of Figure S1a, dividing them into groups with good prognosis (categories 1 and 2, black curve) and worse prognosis (categories 3, 4, and 5, red curve), according to the ISUP grade. Kaplan-Meier curves are significantly different with *p* < 0.0001. Figure S2: SRXN1 expression is increased in advanced PCa and in most prostate tumor cell lines, and its overexpression is associated with poor prognosis and lower disease-/progression-free survival. (A) Levels of SRXN1 expression in wild-type prostate (control) and advanced PCa samples (Gleason scores 8 and 9) from a study available on the GEO profile human database (reference series GSE5016) [1]. (B) SRXN1 gene expression in different prostate cell lines (androgen sensitive and castration-resistant) obtained from a study available on the GEO profile human database (reference series GSE4016) [2]. (C) Expression of SRXN1 (median) in five PCa iClusters generated by the Cambridge Carcinoma of the Prostate App (camcAPP dataset) [3] from an integrative study [4]. iClusters 1 (red), 3 (green), and 5 (orange) represent groups of patients with worse prognosis, while iClusters 2 (blue) and 4 (purple) represent groups with better prognosis. Boxplots are significantly different, with *p* = 5.7833^−9^. (D) Expression of SRXN1 (median) in five PCa iClusters generated by the Cambridge Carcinoma of the Prostate App (camcAPP dataset) [3] from an integrative study [4]. iClusters 1 (red), 3 (green), and 5 (orange) represent groups of patients with worse prognosis, while iClusters 2 (blue) and 4 (purple) represent groups with better prognosis. Boxplots are significantly different with *p* = 0.034473. (E) Expression of SRXN1 (median) in six PCa iClusters generated by the Cambridge Carcinoma of the Prostate App (camcAPP dataset) [3] from an integrative study [5]. iClusters 1 (salmon), 2 (dark yellow), 3 (green), and 4 (turquoise) are groups of patients with more favorable prognosis with minimal copy number alterations (CNA), while iClusters 5 (light blue) and 6 (lilac) include most of the metastatic tumors with substantial CNA. Boxplots are significantly different, with *p* = 3.42^−6^. (F) Kaplan-Meier curve displaying the probability of freedom from biochemical recurrence of PCa with (red) or without (blue) SRNX1 overexpression, cataloged by the Cambridge Carcinoma of the Prostate App (camcAPP dataset) [3] from an integrative study [5]. Curves are statistically different with *p* = 0.0079.
