# Supplementary material for: Sulfiredoxin as a Potential Therapeutic Target for Advanced and Metastatic Prostate Cancer
Source: Oxid Med Cell Longev. 2020 Jan 20;2020:2148562. doi: 10.1155/2020/2148562 (PMC7201699; doi:10.1155/2020/2148562)
Supplement: Supplementary Materials — Table S1: description of the patient clinical data used in the preparation of the TMAs, such as Gleason score, prognostic category, survival time, and patient outcome. Figure S1: patients with PCa grouped by prognosis category (1-5) show expected survival curves. (A) Global survival curve of patients with PCa associated to prognosis category (1-5, from more differentiated to less differentiated), according to the ISUP grade. Clinical data are from those patients which prostate samples were used to construct TMAs. Kaplan-Meier curves are statistically different with p < 0.0001. (B) Additional analysis of the same patients of Figure S1a, dividing them into groups with good prognosis (categories 1 and 2, black curve) and worse prognosis (categories 3, 4, and 5, red curve), according to the ISUP grade. Kaplan-Meier curves are significantly different with p < 0.0001. Figure S2: SRXN1 expression is increased in advanced PCa and in most prostate tumor cell lines, and its overexpression is associated with poor prognosis and lower disease-/progression-free survival. (A) Levels of SRXN1 expression in wild-type prostate (control) and advanced PCa samples (Gleason scores 8 and 9) from a study available on the GEO profile human database (reference series GSE5016) [1]. (B) SRXN1 gene expression in different prostate cell lines (androgen sensitive and castration-resistant) obtained from a study available on the GEO profile human database (reference series GSE4016) [2]. (C) Expression of SRXN1 (median) in five PCa iClusters generated by the Cambridge Carcinoma of the Prostate App (camcAPP dataset) [3] from an integrative study [4]. iClusters 1 (red), 3 (green), and 5 (orange) represent groups of patients with worse prognosis, while iClusters 2 (blue) and 4 (purple) represent groups with better prognosis. Boxplots are significantly different, with p = 5.7833−9. (D) Expression of SRXN1 (median) in five PCa iClusters generated by the Cambridge Carcinoma of the Prostate App (camcAPP da [file 2148562.f1.pdf]

Supplementary Materials

Table S1. Clinical data of the patients whose prostate biopsy was used in the preparation of the tissue microarrays (TMAs).

TMA number	Biopsy number/ Year	Gleason Score	Prognostic Category (ISUP)	Survival time (months)	Outcome / Final Result	IHC Score to SRXN1
PR1	B1375/80	4+4	4		No information	1
PR2	BR2637 + B2686/80	4+4	4		No information	1
PR3		4+4	4	123	Death by disease	1
PR4	B3238/80	4+4	4	123	Death by disease	1
PR5/PR6	B40/81+ B179/81	4+4	4	63	Death by disease	1
PR10	B2734/81	4+4	4	28	Death by disease	1
PR11	B2588/82	4+5	5		No information	1
PR12	B1194/83	4+5	5		No information	1
PR13		4+4	4	32	Death by disease	1
PR15	B1195/85	5+4	5	36	Death by disease	1
PR16	B1469/85	4+4	4		No information	1
PR17	B1882/85	5+4	5		No information	0
PR19	B88/86	4+4	4	26	Death by disease	0
PR20	B3200/86	4+4	4	160	Death by disease	1
PR21		3+3	1		No information	1
PR22	B526/87	4+4	4	49	Death by disease	1
PR23	B640/87	4+3	3		Loss of follow-up	1
PR24	B1771/87	5+5	5		No information	1
PR25	B2019/87	3+3	1	215	Death by disease	0
PR26	B2274/87	5+5	5		No information	0
PR27	B61/88	* 3+4	2		Loss of follow-up	0
PR28	B128/88	3+3	1		No information	1
PR29	B905/89	4+4	4		No information	1
PR30	B1209/89	* 3+4	2		Loss of follow-up	1
PR31	B2172/89	5+5	5	120	Death with disease (another cause)	1
PR32	B4264/89	4+3	3	146	Death by disease	1
PR33	B278/91	3+3	1		Loss of follow-up	1
PR35	B1029/91	3+3	1	159	Death by disease	0
PR36	B2332/91	3+4	2	186	Death by disease	0
PR37	B4613/91	4+4	4	24	Death by disease	1
PR38	B4645/91	3+3	1		Loss of follow-up	1
PR39	B5072/91	3+4	2		No information	1
PR40	B5626/91	4+4	4		Loss of follow-up	1
PR41	B5764/91	* 3+4	2		Loss of follow-up	1
PR42	B5807/91	3+3	1	148	Death without disease	1
PR43	B273/92	* 3+3	1		No information	0
PR44	B1099/92	3+4	2		Loss of follow-up	0

PR45	B1426/92	3+3	1	212	Death without disease	0
PR46	B2480/92	4+3	3		Loss of follow-up	1
PR47	B3810/92	* 3+3	1	146	Death without disease	0
PR48	B4006/92	3+4	2		Death with disease (another cause)	1
PR49	B4101/92	4+4	4	46	Death by disease	1
PR50	B5169/92	4+3	3	8	Death with disease (another cause)	1
PR52	B377/93	4+4	4	20	Death by disease	1
PR53	B1657/93	* 4+3	3		Loss of follow-up	0
PR54	B1805/93	4+3	3		No information	0
PR55	B1935/93	4+3	3		Loss of follow-up	1
PR56	B2108/93	4+4	4	45	Death by disease	1
PR58	B2836/93	5+4	5		No information	1
PR59	B436/94	3+4	2	52	Loss of follow-up	0
PR60	B3616/94	4+3	3		Loss of follow-up	1
PR61	B1551/95	3+3	1		Loss of follow-up	0
PR62	B1749/95	3+4	2		Loss of follow-up	0
PR63	B1885/95	3+3	1		Loss of follow-up	0
PR64	B4371/95	3+4	2		Loss of follow-up	0
PR66	B6420/95	4+3	3	155	Loss of follow-up	0
PR67	B6972/95	4+3	3	43	Death without disease	1
PR68	B432/96	4+3	3	158	Death by disease	0
PR69	B3480/96	4+4	4	28	Death by disease	1
PR70	B3742/96	* 3+3	1		Loss of follow-up	1
PR71	B5387/96	* 3+3	1	215	Live with disease	0
PR72	B891/97	4+3	3		Live without disease - Loss of follow-up in 2008	0
PR73	B1927/97	3+3	1		Live without disease - Loss of follow-up in 2008	1
PR74	B3134/97	4+3	3	117	Death with disease (another cause)	1
PR75		4+5	5		No information	1
PR76	B4198/97	5+3	4	60	Death by disease	1
PR77	B4700/97	3+5	4	73	Death by disease	1
PR78	B6280/97	5+4	5		No information	0
PR79	B6487/97	* 4+3	5	213	Free from disease	0
PR80	B7295/97	5+5	5	38	Death by disease	0
PR81	B7773/97	5+4	5	70	Loss of follow-up	0
PR82	B1417/98	4+4	4	106	Death by disease	1
PR83	B2725/98	3+3	1		Loss of follow-up	1
PR84	B3309/98	3+3	1		No information	1
PR85	B4391/98	4+5	5		Live with disease	0
PR86	B4773/98	* 4+3	3		No information	1
PR87	B5939/98	* 4+3	3	173	Live with disease	1
PR88	B5967/98	4+5	5	29	Death by disease	1
PR89	B6513/98	4+3	3		Loss of follow-up	0
PR90	B7195/98	4+3	3	66	Death by disease	0
PR91	B7964/98	* 3+3	1		Live without disease - Loss of follow-up	0

PR92	B8133/98	3+4	2	178	Free from disease	0
PR93	B3150/99	3+3	1	45	Death by disease	0
PR94		* 3+3	1	103	Death without disease	1
PR95		4+3	3	24	Death by disease	1
PR99	B6870/99	* 3+3	1		Loss of follow-up	0
PR100		3+4	2	173	Death without disease	0
PR101	B6944/99	3+4	2		Loss of follow-up	0
PR102	B7241/99	3+4	2	46	Death by disease	1
PR103	B7418/99	3+3	1		Loss of follow-up	1
PR104		3+3	1	175	Live with disease	1
PR106	B414/2000	3+3	1		Loss of follow-up	0
PR107	B715/00	3+3	1		Loss of follow-up	0
PR108	B2299/00	4+5	5		No information	0
PR109	B3002/00	3+3	1		No information	0
PR110	B3026/00	* 3+3	1	141	Live with disease	0
PR111	B3055/00	3+4	2	111	Live with disease	0
PR112	B3332/00	3+3	1	173	Free from disease	0
PR113	B4884/00	3+3	1	168	Free from disease	1
PR114	B5059/00	* 4+3	3	163	Free from disease	1
PR115	B5394/00	3+3	1		Loss of follow-up	0
PR117	B5967/00	3+4	2	177	Free from disease	0
PR118	B6232/00	3+3	1	112	Free from disease	1
PR119	B6799/00	3+4	2	157	Death without disease	0

* represents samples with adjacent nonneoplastic tissue; ISUP: International Society of Urological Pathology; IHC: Immunohistochemistry

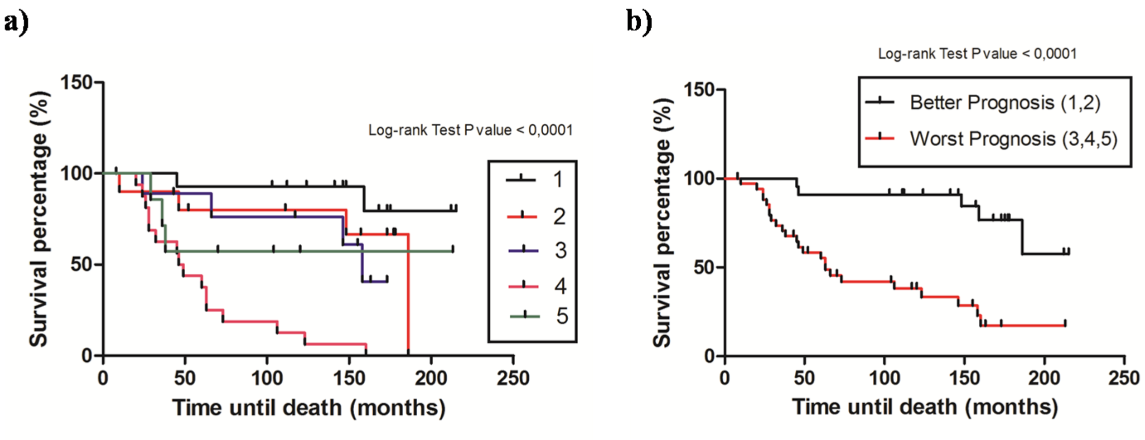

Figure S1. Patients with prostate cancer (PCa) grouped by prognosis category (1-5) show expected survival curves. A) Global survival curve of patients with PCa associated to prognosis category (1-5, from more differentiated to less differentiated), according to International Society of Urological Pathology (ISUP) grade. Clinical data are from those patients which prostate samples were used to construct tissue microarrays (TMAs). Kaplan-Meier curves are statistically different with $p<0.0001$. **B)** Additional analysis of the same patients of Figure S1a, dividing them into groups with good prognosis

(categories 1 and 2, black curve) and worse prognosis (categories 3, 4 and 5, red curve), according to ISUP grade. Kaplan-Meier curves are significantly different with p<0.0001.

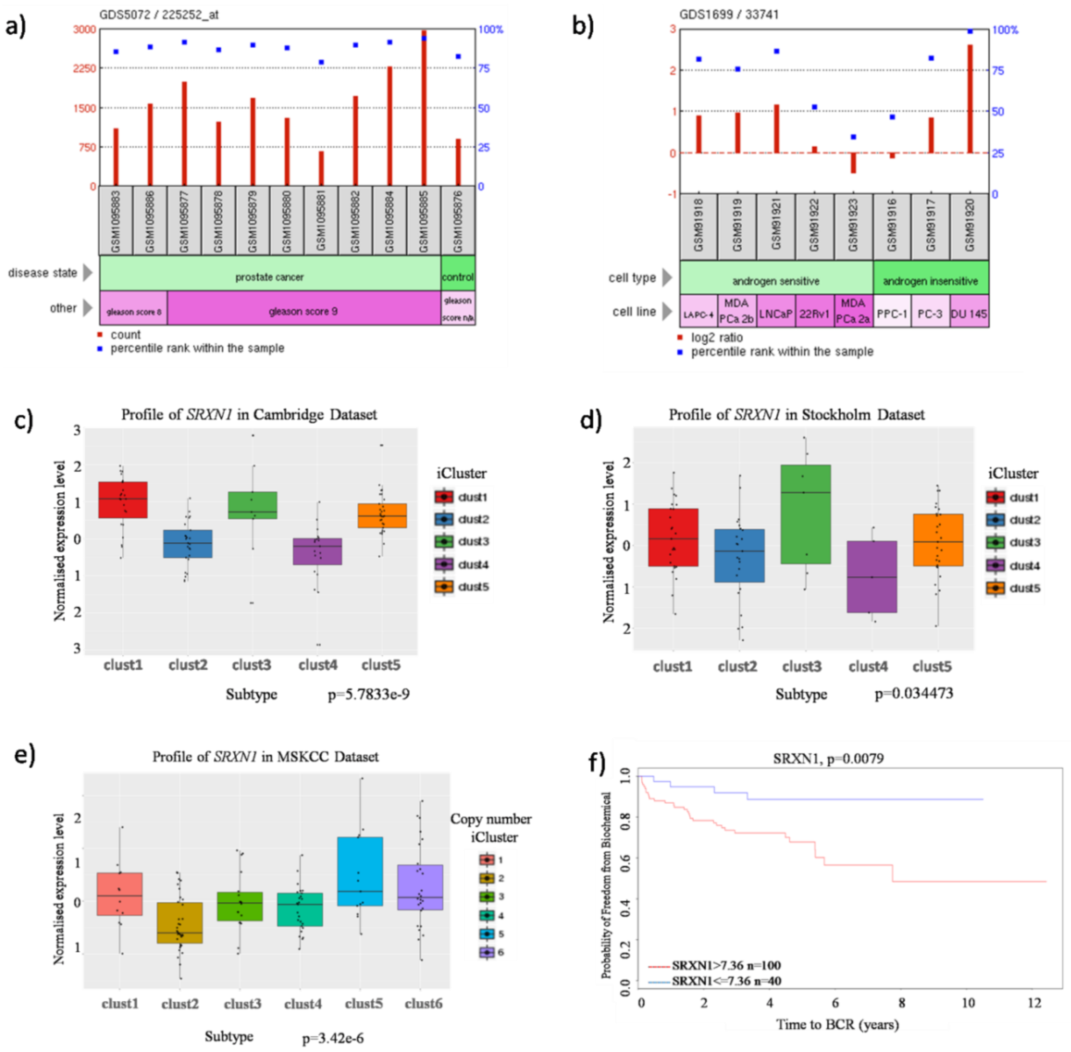

Figure S2. ***SRXN1* expression is increased in advanced prostate cancer (PCa) and in most prostate tumor cell lines, and its overexpression is associated with poor prognosis and lower disease-/progression-free survival. A)** Levels of *SRXN1* expression in wild type prostate (control) and advanced PCa samples (Gleason score 8 and 9) from a study available on the GEO profiles human database (reference series GSE45016) [1]. **B)** *SRXN1* gene expression in different prostate cell lines (androgen sensitive and castration-resistant) obtained from a study available on the GEO profiles human database (reference series GSE4016) [2]. **C)** Expression of *SRXN1* (median) in five PCa iClusters generated by the Cambridge Carcinoma of the Prostate App (CamcAPP dataset) [3] from an integrative study [4]. iClusters 1 (red), 3 (green) and 5 (orange) represent groups of patients with worse prognosis, while iClusters 2 (blue) and 4 (purple) represent groups with better prognosis. Boxplots are significantly different, with $p=5.7833^{-9}$. **D)** Expression of *SRXN1* (median) in five PCa iClusters generated by the CamcAPP dataset [3] from an integrative study [4]. iClusters 1 (red), 3 (green) and 5 (orange) represent groups of patients with worse prognosis, while iClusters 2 (blue) and 4 (purple) represent groups with better prognosis. Boxplots are significantly different with p=0.034473. **E)** Expression of *SRXN1* (median) in six PCa iClusters generated by the CamcAPP dataset [3] from an integrative study [5]. iClusters 1 (salmon), 2 (dark yellow), 3 (green) and 4 (turquoise) are groups of patients with more favorable prognosis with minimal copy number alterations (CNA), while iClusters 5 (light blue) and 6 (lilac) include most of the metastatic tumors with substantial CNA. Boxplots are significantly different, with

$p=3.42^{-6}$. **F)** Kaplan-Meier curve displaying the probability of freedom from biochemical recurrence of PCa patients with (red) or without (blue) *SRNX1* overexpression, cataloged by the CamcAPP dataset [3] from an integrative study [5]. Curves are statistically different with p=0.0079.

References

[1] T. K. Satake H Furihata M, Anchi T, Sakoda H, Kawada C, Iiyama T, Ashida S, Shuin T, “The ubiquitin-like molecule interferon-stimulated gene 15 is overexpressed in human prostate cancer,” *Oncol. Rep.*, vol. 23, no. 1, pp. 11–16, 2010.

[2] H. Zhao *et al.*, “Genome-wide characterization of gene expression variations and DNA copy number changes in prostate cancer cell lines,” *Prostate*, vol. 63, no. 2, pp. 187–197, 2005.

[3] M. J. Dunning *et al.*, “Mining Human Prostate Cancer Datasets: The "camcAPP" shiny app,” *EBioMedicine*, vol. 17, pp. 5–6, Mar. 2017.

[4] H. Ross-Adams *et al.*, “Integration of copy number and transcriptomics provides risk stratification in prostate cancer: A discovery and validation cohort study,” *EBioMedicine*, vol. 2, no. 9, pp. 1133–1144, Sep. 2015.

[5] B. S. Taylor *et al.*, “Integrative Genomic Profiling of Human Prostate Cancer,” *Cancer Cell*, vol. 18, no. 1, pp. 11–22, Jul. 2010.
